# 

**Published:** 1892-04-30

**Authors:** 


### CONTENTS.

**The Sunlight Cure** - - - - - - - - - - - 65

**Passing Topics.**—In Reversion - - - - - - - - 66

**The Doctor's Armchair.**—With Modern Eyes - - - - - 67

**Poisons and Poisoners.**—Strychnia—Dr. Palmer - - - 68

**Medical History from the Earliest Times.** By E. T. Withington, M.A., M.B.Oxon. VI.—Hindoo Medicine - - 69

**The Hospital Boom** - - - - - - - - - - - 70

**Annotations.**—The Clergy and Vivisection—Mr. Goschen and Doctors' Incomes—Ladies' Chins - - - - - - - 71

**Notes and News** - - - - - - - - - - - 72

**Around the Hospitals** - - - - - - - - - 73

**Scraps and Gleanings** - - - - - - - - - 73

**The Practitioner's Mirror and Retrospect—**

Medicine.—Amœbic Dysentery - - - - - - - - 74

Surgery.—The Question of Surgical Interference in Cases of Perityphlitis - - - - - - - - - - - - 74

**The Institutional Workshop—**

Within the Hospitals. I.—Royal Berkshire Hospital, Reading - - - - - - - - - - - - - 76

Practical Points - - - - - - - - - - - - 77

Hospital Construction.—The Royal South London Ophthalmic Hospital - - - - - - - - - - - - - 78

**The Student of Medicine.** — Appointments — Vacancies—Pass Lists.

### EXTRA SUPPLEMENT.—THE NURSING MIRROR.

**En Passant.**—The Co-operative System—Bygone Uniforms—Observant Womankind—A Pleasing Comparison—Lerwick—The Royal British Nurses' Association—Nurses and Religion—Short Items—Nursing at the Royal Free - - - xxix

**Ventilation, Disinfection, and Diet.** By P. Caldwell Smith, M.D. III.—Pure Air and Natural Ventilation - xxx

**The Nursing of the Insane.** By A Medical Officer. I.—Why Training is Necessary - - - - - - - - - xxxi

**For Reading to the Sick.**—"The Point of View" - - xxxi

**Nurses for the English in India** - - - - - - - xxxii

**Technical Teachers** - - - - - - - - - - xxxii

**Presentation** - - - - - - - - - - - - xxxii

**Nursing Uniforms.** V.—Outdoor Uniforms - - - - xxxiii

**Everybody's Opinion.**—The Nurses' Co-operation—Male Probationers—Would-be Suicides—A Walking Tour - - xxxiii

**Appointment—Notes and Queries** - - - - - - - xxxiii

**Nursing Homes.** II.—St. Thomas's Hospital. III.—Westminster - - - - - - - - - - - - - - xxxiv

**Among the Institutions** - - - - - - - - - xxxiv

**Four Months in a Hospital Ward** I.—A Personal Experience in a Provincial Hospital - - - - - - xxxv

**A True Story of Flowers** - - - - - - - - - xxxvi

